# Practical Considerations before Installing Ground-Based Geodetic Infrastructure for Integrated InSAR and cGNSS Monitoring of Vertical Land Motion

**DOI:** 10.3390/s17081753

**Published:** 2017-07-31

**Authors:** Amy L. Parker, Will E. Featherstone, Nigel T. Penna, Mick S. Filmer, Matthew C. Garthwaite

**Affiliations:** 1 Department of Spatial Sciences and the Institute for Geoscience Research, Curtin University of Technology, GPO Box U1987, Perth 6845, Australia; amy.parker@curtin.edu.au (A.L.P.); m.filmer@curtin.edu.au (M.S.F.); 2 School of Engineering, Newcastle University, Newcastle upon Tyne NE1 7RU, UK; nigel.penna@newcastle.ac.uk; 3 Geodesy Section, Community Safety and Earth Monitoring Division, Geoscience Australia, GPO Box 378, Canberra 2601, Australia; matt.garthwaite@ga.gov.au

**Keywords:** vertical land motion, InSAR, corner reflectors, continuous GNSS, TerraSAR-X, sentinel-1, geodetic networks, displacement monitoring

## Abstract

Continuously operating Global Navigation Satellite Systems (cGNSS) can be used to convert relative values of vertical land motion (VLM) derived from Interferometric Synthetic Aperture Radar (InSAR) to absolute values in a global or regional reference frame. Artificial trihedral corner reflectors (CRs) provide high-intensity and temporally stable reflections in SAR time series imagery, more so than naturally occurring permanent scatterers. Therefore, it is logical to co-locate CRs with cGNSS as ground-based geodetic infrastructure for the integrated monitoring of VLM. We describe the practical considerations for such co-locations using four case-study examples from Perth, Australia. After basic initial considerations such as land access, sky visibility and security, temporary test deployments of co-located CRs with cGNSS should be analysed together to determine site suitability. Signal to clutter ratios from SAR imagery are used to determine potential sites for placement of the CR. A significant concern is whether the co-location of a deliberately designed reflecting object generates unwanted multipath (reflected signals) in the cGNSS data. To mitigate against this, we located CRs >30 m from the cGNSS with no inter-visibility. Daily RMS values of the zero-difference ionosphere-free carrier-phase residuals, and ellipsoidal heights from static precise point positioning GNSS processing at each co-located site were then used to ascertain that the CR did not generate unwanted cGNSS multipath. These steps form a set of recommendations for the installation of such geodetic ground-infrastructure, which may be of use to others wishing to establish integrated InSAR-cGNSS monitoring of VLM elsewhere.

## 1. Introduction and Background

Vertical land motion (VLM), i.e., subsidence or uplift, arising from natural (e.g., tectonic) and/or anthropogenic (e.g., subsurface resource extraction) phenomena affects geodetic benchmark heights and complicates the sea level record at tide gauges [1,2]. Earth-orbiting artificial satellites, including Global Navigation Satellite Systems (GNSS, notably GPS) [3] and/or Interferometric Synthetic Aperture Radar (InSAR) [4,5,6] can be used to measure and monitor VLM. Whilst these techniques are complementary, there are two key differences that need to be considered when integrating these different types of measurements.

The first key difference is that GNSS provides estimates of VLM in terms of ellipsoidal heights at discrete points, whereas InSAR provides estimates of range change in the satellite’s slanted line of sight (LoS) on regional scales (10 s to 100 s of km) at spatial resolutions reaching <10 m. The LoS of a SAR satellite is most sensitive to VLM, due to the usually steep incidence angle of the sensor, but the near-polar orbital plane of SAR satellites means that it also contains any motion in the east-west and, to a much lesser extent, north-south directions. Therefore, to directly compare/integrate the two measurement types, either the 3D GNSS coordinates should be mapped into the 1D InSAR LoS, or the vertical component of motion should be extracted from the LoS InSAR measurement. The latter may be achieved by transforming all LoS displacements into the vertical direction under the assumption that no relative horizontal motion is occurring [7]; through combination of InSAR measurements made from different satellite geometries or “look” directions (e.g., ascending versus descending orbits [8]); or through removal of any relative horizontal motion determined by GNSS [9,10]. We assume hereafter that one of these approaches has been adopted and therefore we are measuring only VLM with both techniques.

The second key difference is that GNSS provides VLM in an absolute sense with respect to some defined reference frame [11,12,13,14], whereas InSAR provides VLM estimates relative to the time of the first SAR acquisition and a local spatial reference within the imaged area. This may be the mean VLM of all pixels in the image [15], or a far-field point or reference region in the images, where it is assumed that no VLM is occurring [16,17]. Alternatively, other geodetic observations, such as GNSS, may be used to constrain VLM at reference point(s), and to convert relative InSAR-measured VLM to absolute VLM in a global reference frame [18,19,20].

In order to make precise comparisons between these two measurement types and thus fully integrate InSAR and GNSS, a ground network of artificial InSAR corner reflectors (CRs) can be deliberately co-located with continuous GNSS (cGNSS). The CRs provide a high-quality, temporally stable phase response in SAR imagery, which is required for reliable InSAR time series of VLM [21]. The deliberate co-location with cGNSS ensures that there are coherent InSAR image pixels close to the cGNSS, and that the position of the cGNSS can be located in the InSAR image. The co-location also avoids the need to use the average of groups of pixels around the cGNSS station under the assumption that they represent the same VLM [22,23]. Any differential VLM between the CR and cGNSS should be monitored via regular repeat differential levelling and used to calibrate InSAR VLM rates. This configuration is particularly advantageous when attempting to measure small-magnitude VLM (e.g., mm/year rather than cm/year).

There is limited published literature on monitoring VLM using deliberately co-located cGNSS and CRs. Studies that do combine InSAR and cGNSS to measure VLM tend to do so without the use of CRs [9,18,24,25], and many InSAR-based studies that have deployed CRs do so primarily to increase the spatial coverage of coherent pixels, without the use of cGNSS [26,27,28,29,30,31,32,33]. Fu et al. [34] validated displacement measurements from such a CR network in China with episodic GPS measurements made at the CR locations over a 140 day period, but no information was given on the distance between the GPS and CR. Other recent applications of CRs and GPS have focused on absolute positioning of CRs, with relative cGNSS [20] or relative GPS [19] used to determine the precise locations of CRs in 2D or 3D, respectively. Larger-scale (over 10,000 s km^2^) CR-GNSS networks include 40 CRs co-located (within 50 m) with episodic-occupation GNSS stations throughout the Surat Basin, Australia [35], and national-scale initiatives to monitor VLM, where the use of co-located cGNSS and CRs is in varying stages of development [36,37,38].

Here we use the example of Perth, Australia, to describe a series of tests conducted before new permanent installations of co-located ground infrastructure to support integrated cGNSS and InSAR monitoring of VLM. In documenting these efforts, we aim to provide some framework for efficiently establishing operational integrated satellite-based VLM monitoring that may be applicable elsewhere. In Perth, InSAR and cGNSS, coupled with repeat levelling over many 10 s of km (not described in this study), are currently being used to measure, map and monitor subsidence caused principally by groundwater extraction [39,40,41]. Once sufficient data has been collected from the network of ground infrastructure described here, the measurements will be integrated in future studies to determine VLM in a global or regional reference frame. The improvements to geodetic infrastructure resulting from this study will help to overcome the challenges identified by Featherstone et al. [39] of measuring small magnitude (<7 mm/year) subsidence, and avoid the need to make further inferences about the magnitude of VLM occurring at the Fremantle tide gauge [40].

## 2. Ground Infrastructure for Measuring VLM in Perth

**Figure 1:**
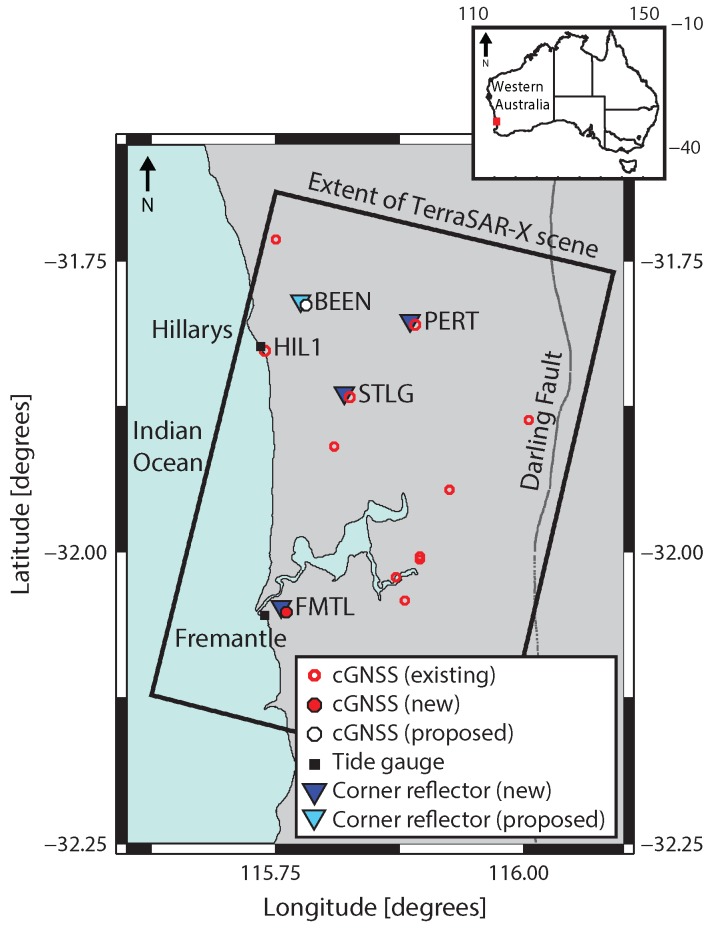
The distribution of ground-based geodetic infrastructure for measuring vertical land motion (VLM) in the Perth region, including: new, proposed and existing continuous GNSS (cGNSS); corner reflectors (new and proposed); and existing tide gauges. The rectangle shows the extent of TerraSAR-X InSAR scenes. The extent of the Sentinel-1B InSAR scenes covers the entire region. STLG—Stirling; PERT—Perth; FMTL—Fremantle; BEEN—Beenyup wastewater injection plant; HIL1—Hillarys. Inset: Map of Australia with the study region highlighted by the red rectangle.

The Perth region hosts a mixture of existing cGNSS infrastructure (Figure 1). The cGNSS stations we discuss in this study are those labelled on Figure 1, and detailed in Table 1.

This includes two cGNSS (PERT and STLG) that are on deep-seated (~1.5 m) pillars, a new cGNSS co-located with a tide gauge at Fremantle (FMTL), and HIL1, that is also located on a tide gauge. (A fifth cGNSS site, labelled BEEN in Figure 1, is soon to be under construction and is therefore omitted from Table 1.) The remaining cGNSS stations in Figure 1 are installed on buildings that can sometimes be subject to changes in height unrelated to VLM, e.g., settlement or thermal expansion and contraction. These commercial installations were never intended to monitor VLM, but instead to provide real-time kinematic GNSS corrections to fee-paying users.

Prior to the work described here, there were no artificial CRs deployed in the Perth region, so previous InSAR studies exploited naturally occurring distributed scatterers from buildings, roads and natural surfaces [41]. During the period of this study, which began in October 2015, SAR imagery over the Perth region was acquired by two satellite missions: X-band Stripmap mode images from TerraSAR-X (TSX: [42]) operated by the German Aerospace Centre (DLR; see Acknowledgements); and C-band Interferometric Wide Swath mode images from the European Space Agency (ESA) Sentinel-1B satellite [43], which began to collect imagery in September 2016. The CRs we use here are triangular trihedral designs of 1 m inner-leg dimension and have been previously characterised during prototype design experiments, where they were shown to be suitable for both X- and C-band SAR data [44].

The cGNSS and CR co-locations resulting from this study are listed in Table 2. This includes the installation of a CR at an existing IGS (International GNSS Service; [45]) cGNSS station PERT (Figure 1 and Table 1), and a commercially operated cGNSS station at Stirling (STLG in Figure 1 and Table 1). A new cGNSS and co-located CR have been installed at Fremantle (FMTL in Figure 1 and Table 1) at a distance of ~1.5 km from the tide gauge, which is—to the best of our knowledge—the first cGNSS-CR-tide-gauge co-location globally, or certainly in Australia [46]. Finally, a proposed co-located cGNSS and CR installation has been tested and is now awaiting construction at the Beenyup wastewater plant (BEEN in Figure 1), which is to inject treated wastewater into subsurface aquifers.

## 3. Practical Considerations for Installing Geodetic Infrastructure

### 3.1. General

The installation of permanent ground infrastructure for any satellite-based VLM monitoring requires sites that (i) have unobstructed sky visibility down to 10 degrees for cGNSS and in the satellite LoS for CRs, (ii) are secure against theft and/or vandalism, (iii) have the landowner’s permission, and (iv) can host ground monuments that, ideally, are connected to bedrock. In sedimentary basins such as in Perth, however, bedrock is not always close to the ground surface at sites that satisfy items (i–iii), so deep-seated foundations have had to be used instead.

### 3.2. cGNSS

Mature, tried and tested standards and recommended practices (SARPs) for the installation of cGNSS have been compiled by numerous national government or international agencies [47]. In Australia, these are promulgated by the Intergovernmental Committee on Surveying and Mapping [48]. These SARPs were followed during the test and permanent cGNSS installations.

Our particular concern was the potential of multipath (reflected signals) caused by the co-located CR, which is a deliberately designed reflecting object. Characterisation of carrier-phase multipath at cGNSS sites is not only non-trivial, but also can adversely affect VLM rates determined from the daily ellipsoidal height time series [49,50]. Multipath prevails in the post-fit carrier-phase residuals, which are the difference between the calculated and observed values [51,52].

We desire to avoid multipath from the CR, but achieve a close enough co-location to allow for regular short-range repeat levelling (following standards outlined in [53]) to monitor any differential VLM between the cGNSS and CR. We therefore took a two-pronged approach to multipath management: (1) locating each CR at least 30 m away (greater than the 20 m specified in [48]) from the cGNSS and out of visual line of sight, subject to the SAR pre-analyses described in Section 3.3; and (2) carrying out temporary test GNSS and CR co-locations, followed by analysis of the zero-difference ionosphere-free carrier-phase (LC) residuals and ellipsoidal height time series from the precise point positioning (PPP) GPS technique [54]. We compared the mean ellipsoidal heights and the average of the daily RMS of the carrier-phase (LC) residuals, measured during these co-location tests, to that measured over an equal time-period directly before and directly after the test (for test epochs, see Table 2). From this, we assessed whether there were changes in ellipsoidal heights or LC carrier-phase residuals that would be indicative of increased multipath.

We chose the PPP technique over a network solution (which outputs double difference (DD) carrier-phase residuals) because in the latter, it not possible to discriminate which site is generating the multipath. We used the NASA JPL GIPSY software version 6.4 for the daily processing, with JPL fiducial-free ’repro 2.1’ orbits and satellite clocks held fixed, a 10 degree elevation angle cut-off, correcting for satellite and receiver phase centre variations using the IGS08 models, modelling tidal ground displacements according to the IERS Conventions 2010 [55], and estimating tropospheric zenith wet delays and horizontal gradients every 5 min, applying the VMF1 tropospheric mapping function [56]. Zenith wet delay and horizontal gradients were estimated using process noise values of 3 mm/√hr and 0.3 mm/√hr, respectively, and ambiguities were fixed to integers, with the coordinates represented in the IGS08 reference frame after transforming the fiducial-free outputs using JPL ’repro 2.1’ daily Helmert (seven) transformation parameters.

This GIPSY-based PPP GPS analysis procedure has been used for monitoring small geophysical movements of a few mm per year [40,57,58]. The quality of PPP coordinate solutions is commensurate with DD relative solutions: for example, [59] describe the computation of a GPS velocity field for the Mediterranean region using the GIPSY (in PPP mode), Bernese (in DD mode) and GAMIT (in DD mode) softwares, and found that the RMS difference from the combined solution of both the DD and PPP solutions were 0.20–0.25 mm/year, based on nearly 900 stations.

### 3.3. InSAR CRs

In addition to the general considerations (items i–iv in Section 3.1), there are additional steps that should be taken when selecting a suitable position for installation of CRs for InSAR. CRs provide high-intensity radar reflections in SAR imagery that are visible above the level of background signal (or “clutter”), thus maintaining a high signal-to-clutter ratio (SCR) over time and facilitating interferometric phase measurements with low variability and a high signal-to-noise ratio [21,44].

In an urban monitoring network, it is inevitable that some sites are located in areas with higher levels of background clutter due to other man-made structures. In these cases, the accompanying CR should be positioned where background scattering is as low as possible, whilst still adhering to the landowner’s conditions, to ensure that the reflection from the CR can be identified correctly with a high SCR [31]. Such sites can be identified by visual pre-analysis of SAR backscatter intensity images, which are calculated from the level 1 single-look complex images provided by the space agencies. Once the most suitable CR test site is selected for each co-location, a temporary CR can be deployed on a wooden palette for two or more SAR satellite passes.

When deployed, the CR must be orientated such that the azimuth and elevation of the boresight vector (originating from the intersection of the three triangular plates) is directed in the SAR satellite’s LoS. This can be achieved using the freely available, physics-based Systems Tool Kit modelling environment [60], and the procedures described in [61], to account for the orbit and look angle of the SAR sensor, and the location of CR deployment. Orientation parameters were initially calculated for descending (north to south) passes of TSX, which was the only SAR satellite operating over Perth at the beginning of this study. However, TSX and Sentinel-1B, which became available in September 2016, have a similar orbital configuration (i.e., right-looking SAR instrument in a descending pass) and look angles (~32° to ~36°), therefore the orientation parameters are within the alignment accuracy (few degrees [61]) and are applicable to descending passes of both missions.

Following the test CR installations, intensity images were analysed to assess the suitability of the CR deployment. For each site and satellite, we calculated a time series of the SCR at the location of the CR using a set of co-registered intensity images. For each image, the SCR is calculated by comparing the backscatter intensity in a window containing the CR response (4 × 4 pixels for TSX; 2 × 2 pixels for Sentinel-1B) relative to the backscatter intensity from surrounding scatterers in a 15 × 15 pixel window [30]. A test deployment is deemed successful if the SCR for TSX is of the order of 30 dB, a level considered to be acceptable for calibration purposes [62], and the CR is then permanently installed. If the test deployment is unsuccessful, the temporary CR is redeployed in another location and these tests repeated until a suitable site is found.

**Figure 2:**
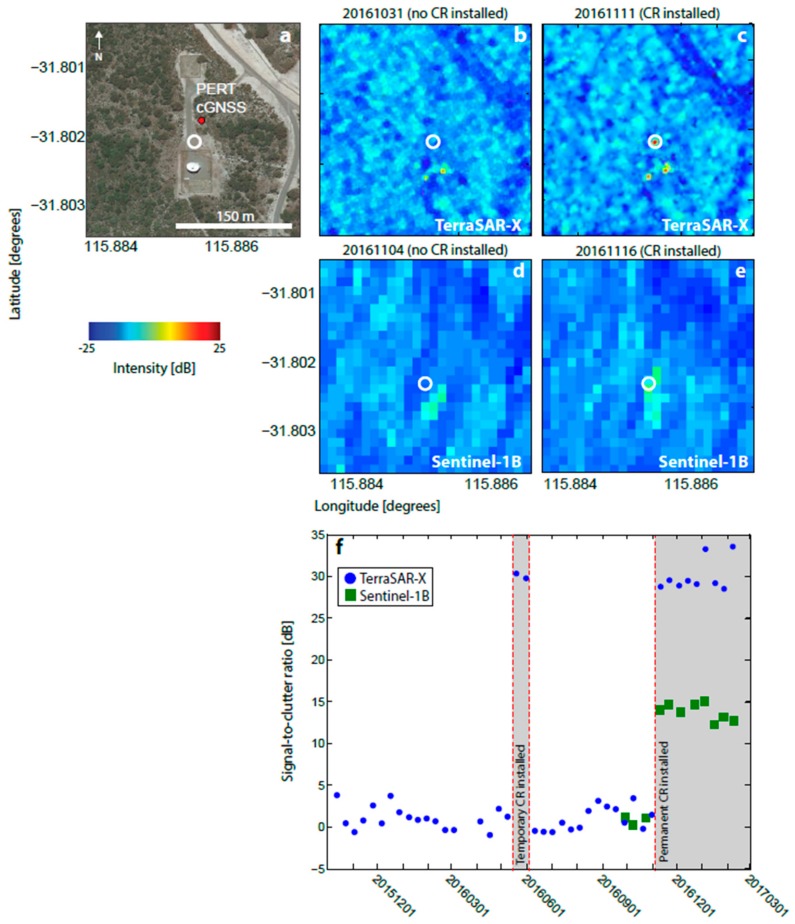
Corner reflector (CR) installation at the existing cGNSS site PERT (location shown in Figure 1). (**a**) Google Earth view of the site showing the cGNSS (red dot) and location of the CR (white circle). (**b**) TSX georeferenced backscatter intensity image prior to CR installation. (**c**) Same as for (b) but after CR installation. (**d**) Sentinel-1B georeferenced backscatter intensity image prior to CR installation. (**e**) Same as for (d), but after CR installation. (**f**) Time series of signal-to-clutter ratio (SCR). Red lines and grey boxes indicate times when the CR was deployed.

The brightness of the CRs is greater in TSX than Sentinel-1B imagery (e.g., see Figure 2) due to the higher radar frequency [44] and higher spatial resolution (5 m in slant range and 20 m in azimuth range for Sentinel-1B, compared to 1.2 m in slant range and 3.3 m in azimuth range for TSX). Similarly, variations in environmental conditions over time due to, e.g., precipitation, are expected to cause trends in clutter [44], but have differing effects on the two satellites due to the difference in the radar frequencies and thus spatial resolutions with respect to the CR and surrounding background scatterers [30]. However, these effects are not significant for the purposes of this study and we do not discuss them further here.

## 4. cGNSS and CR Co-Locations

### 4.1. New CRs at Existing cGNSS Sites: Perth and Stirling

The first CR test installations were made at two existing cGNSS sites: Perth and Stirling (PERT and STLG in Figure 1 and Table 1), as detailed in Table 2. In both cases, the CR was temporarily deployed for two overpasses of TSX (~20 days). Figure 2 shows backscatter intensity images at PERT for the CR located 34 m from the cGNSS. This site has few other man-made structures, therefore low levels of background clutter (−12 dB on average for TSX over the area shown in Figure 2b before temporary CR deployment). Consequently, the CR is clearly visible in the intensity imagery, with SCRs of ~30 dB for TSX and ~13 dB for Sentinel-1B.

**Figure 3:**
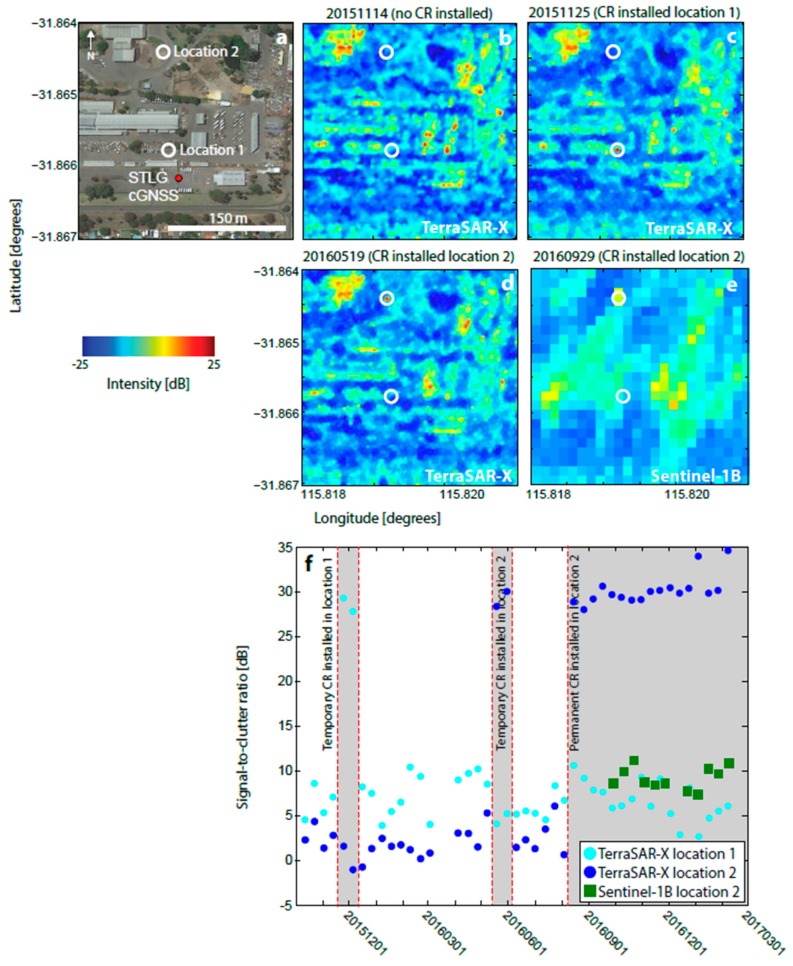
Corner reflector (CR) installations at the existing cGNSS at Stirling (STLG in Figure 1). (**a**) Google Earth view of the site showing the cGNSS (red dot), the initial higher-clutter CR site (white circle labelled location 1) and permanent CR location (white circle labelled location 2). (**b**) TSX georeferenced backscatter intensity image prior to installation. (**c**) Same as for (b) but after the CR installation at location 1. (**d**) Same as for (b,c), but after the CR was installed at the permanent location. (**e**) Sentinel-1B georeferenced backscatter intensity image after permanent CR installation. (**f**) Time series of signal-to-clutter ratio (SCR) at locations 1 and 2. Red lines and grey boxes indicate times when the CR was deployed.

Unlike PERT, STLG is located in an environment with higher levels of background clutter (Figure 3a). Therefore, selecting a suitable, low clutter site that was reasonably close to the cGNSS, but did not impact upon the landowner, was more problematic. The initial test location (location 1 in Figure 3a,c) was 46 m away from the cGNSS, but had a background clutter value of −8 dB for TSX, 33% larger than that at PERT. Consequently, the phase response from the CR was considered to be cluttered by the response from surrounding scatterers.

A second test deployment (location 2 in Figure 3a,d,e) was carried out 196 m from the cGNSS, but in a less cluttered area (background TSX clutter value of −12 dB) where the CR could be more easily identified in the intensity imagery. This location offered a small improvement, with the time series in Figure 3f showing that the SCR for TSX increased by ~20 dB for a CR placed at location 1, and by ~25 dB at location 2. The SCRs for TSX and Sentinel-1B for the permanent installation at location 2 are ~30 dB and ~11 dB, respectively.

**Figure 4:**
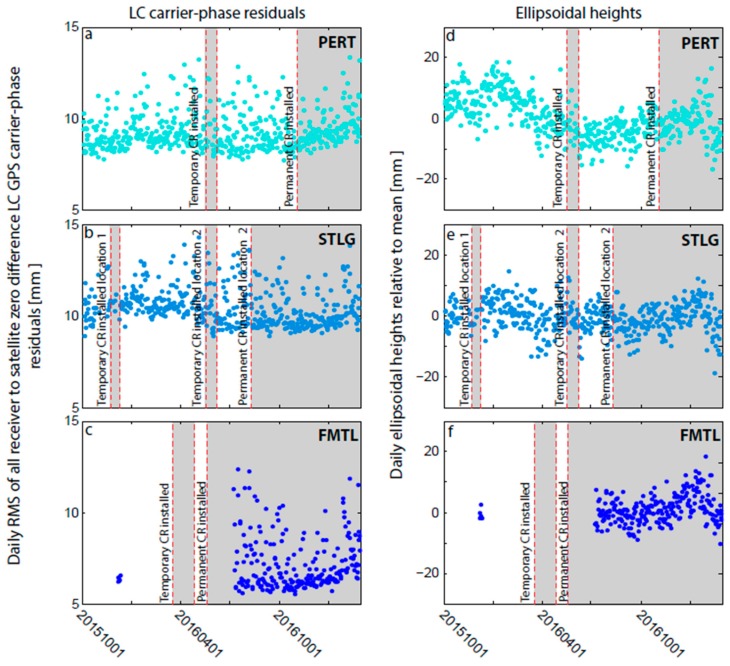
(**a**–**c**) Daily RMS of all receiver to satellite zero-difference ionosphere-free carrier-phase (LC) residuals (mm) calculated from static PPP processing using the NASA JPL GIPSY software (version 6.4). (**d**–**f**) Daily ellipsoidal height time series (mm) from the same PPP processing used for (a–c). Red lines and grey boxes indicate times when the accompanying corner reflector (CR) was temporarily/permanently deployed at each cGNSS site (Table 2).

Time series of ellipsoidal heights and LC carrier-phase residuals at STLG and PERT indicated that the CRs did not cause detectable LC carrier-phase GPS multipath at these two sites (Figure 4). The mean GPS ellipsoidal heights when the CRs were in place are commensurate with those measured over the same time period directly before and after the CR was installed (Table 3), all falling within a range of 7 mm at PERT and 4 mm at STLG, and commensurate with the overall height time series standard deviations of 7 mm and 5 mm for PERT and STLG, respectively. These insignificant differences were further confirmed by a t-test. The average of the daily RMS of the LC carrier-phase residuals when the CR is in place are also commensurate with the mean measured over the same time period directly before, and directly after the CR was installed (Table 3). 

In light of these co-location tests, the CR installations at PERT and STLG (location 2) were made permanent by bolting the reflector to a specially constructed concrete foundation. Each foundation is one-metre square with a 0.6 m central core attached to metal reinforcing rods driven into the ground until refusal (~2 m) for added stability. Local levelling ties between the CR and cGNSS at both PERT and STLG were completed in October 2016 to first-order levelling standards, where the two-way levelling disclosure must be <2√d, where d is the distance of the traverse length between benchmarks [53].

We would expect any strong generator of multipath to manifest in the ellipsoidal heights and LC carrier-phase residuals within 24 h of CR installation, and certainly within the ~20-day CR test periods used in Table 3. This is further supported by comparison of the average of the daily RMS of the LC carrier-phase residuals in the 5, 10, 20 and 40 days directly before the permanent installation to the 5, 10, 20 and 40 days directly after the permanent installation, respectively (Table 4). Regardless of the time window chosen, we observe no detectable increase in the average RMS of the carrier-phase residuals. This vindicates our >30 m separation between the CR and cGNSS and placing them out of visual line of sight, but we suggest readers carry out more comprehensive multipath testing if one, or both of these conditions are not met.

### 4.2. New Co-Located cGNSS and CR Site: Fremantle

The long-recording (since 1897) Fremantle tide gauge was not previously co-located with a cGNSS (Figure 1), and assuming that VLM at the tide gauge is identical to that at the 32-km-distant cGNSS at PERT is questionable [40]. Ideally, a cGNSS should be placed in very close proximity to a tide gauge so that the derived VLM is representative of that at the tide gauge [63], which allows for the separation of any VLM from the tide gauge record. However, the Fremantle tide gauge is situated next to the Western Australian Maritime Museum building, masking a large proportion of the sky for both GNSS and SAR satellites, and whose architecture makes it an extremely strong generator of GNSS multipath. Additionally, potential sources of electrical interference to GNSS come from communications towers nearby to support the shipping port’s operations.

**Figure 5:**
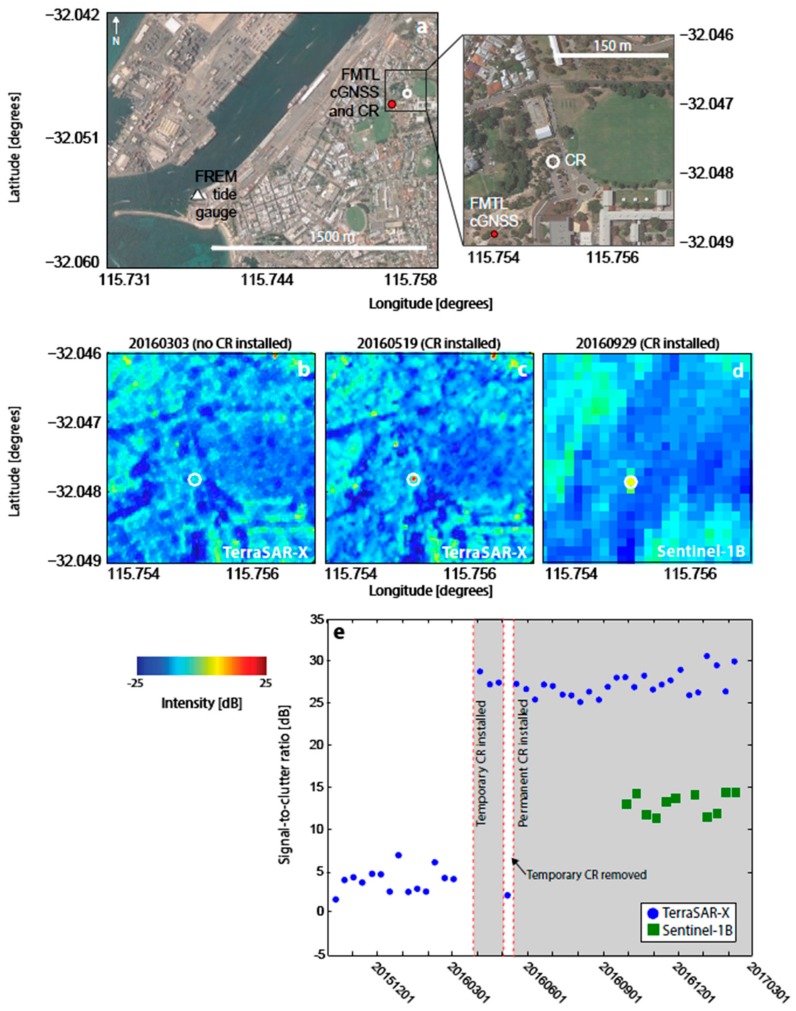
Corner reflector (CR) installation at John Curtin College of the Arts, ~1.5 km from the Fremantle tide gauge (labelled FMTL in Figure 1). (**a**) Google Earth view of the site showing the location of the tide gauge (white triangle), cGNSS (red dot) and CR location (white circle). Inset: enlarged view of the site showing the same area as (b–d). (**b**) TSX georeferenced backscatter intensity image prior to CR installation. (**c**) Same as for (b) but after CR installation. (**d**) Sentinel-1B georeferenced backscatter intensity image after CR installation. (**e**) Time series of signal-to-clutter ratio (SCR) at the location of the CR. Red lines and grey boxes indicate times when the CR was deployed.

Finding a suitable site proximal to the tide gauge proved problematic in the developed town of Fremantle. Numerous sites were rejected for one, more or all of the reasons covered in Section 3.1. A potentially suitable site was found in the grounds of a school ~1.5 km away from the tide gauge (Figure 5a), far closer to the tide gauge than PERT (Figure 1), and therefore much easier to conduct first-order levelling connections.

**Figure 6:**
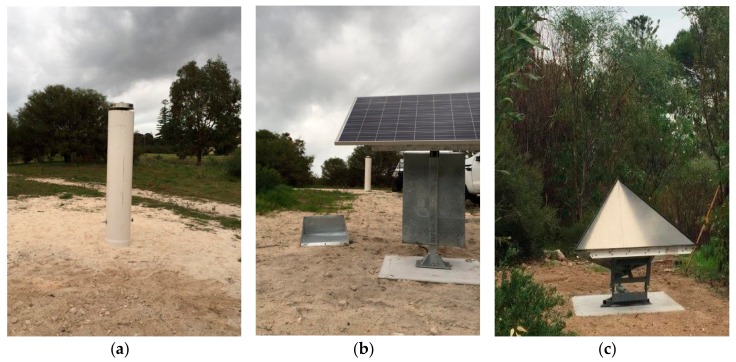
The co-located permanent cGNSS and corner reflector (CR) installations at FMTL (Figure 1 and 5): (**a**) two-metre-tall pillar hosting a Dorne-Margolin-type choke-ring antenna, (**b**) solar panel and padlocked housing beneath for the GNSS receiver, batteries and 4G modem, and (**c**) the CR on a concrete foundation. Images courtesy of Ken Leighton.

Priority was first given to identifying a secure location for the cGNSS within the school grounds, and a test GNSS installation was carried out 7–11 December 2015. Analysis of the PPP GPS LC carrier-phase residuals showed this site to be cleaner than PERT or STLG (Table 3), the average RMS being 7.2 mm. Following the successful GNSS test deployment, TSX backscatter intensity imagery was used to identify prospective sites in the school grounds with low background clutter for the temporary CR deployment (Figure 5). A prospective site was selected with background clutter levels of −10 dB, and that was offset from the proposed cGNSS site by 122 m, and not inter-visible because of vegetation (Figure 6c). The temporary CR was then deployed for three overpasses of TSX (Table 2) and the intensity images re-analysed, showing the site to be suitable (Figure 5b–e). The SCRs calculated for TSX and Sentinel-1B (available ~six months after permanent CR deployment) at this site are ~26 dB and ~14 dB, respectively.

We permanently installed the cGNSS (named FMTL: Figure 1, Table 1, Figure 6a) and CR (Figure 6c), using the same CR foundation construction as for PERT and STLG. A zero-epoch first-order levelling traverse (following the procedures described in Section 4.1) was completed in May 2017, which connected the FMTL cGNSS, its reference/witness marks, four monuments on the concrete foundation upon which the CR is mounted, and the Fremantle tide gauge. The placement of three witness marks for all sites is largely the same, each being placed at a bearing of 120° and 3 m from the cGNSS antenna. It is planned that levelling surveys here and at all other co-located CR and cGNSS sites will be repeated at least annually, specifically at the same time of year with the same equipment and field procedures so as to reduce any residual systematic errors.

### 4.3. Proposed Co-Located cGNSS and CR Site: Beenyup

A new (fourth) co-located cGNSS and CR installation is soon to be constructed at the Beenyup wastewater treatment plant (BEEN in Figure 1), which is to inject ~28 GL/year of potable water into subsurface aquifers. Recent observations linking wastewater injection to ground uplift made in the United States [64] set a precedent for monitoring possible VLM that may be linked to managed aquifer recharge at this site, and the residual effect, if any, upon broader regional subsidence in Perth caused by groundwater extraction.

We repeated our series of site-suitability experiments, carrying out a simultaneous GNSS and CR test deployment. A temporary GNSS receiver was installed 12–15 December 2016. The average of the four days of PPP GPS LC carrier-phase residuals was 10.8 mm, commensurate with the values for PERT and STLG (Table 3), but noisier than FMTL (7.2 mm).

**Figure 7:**
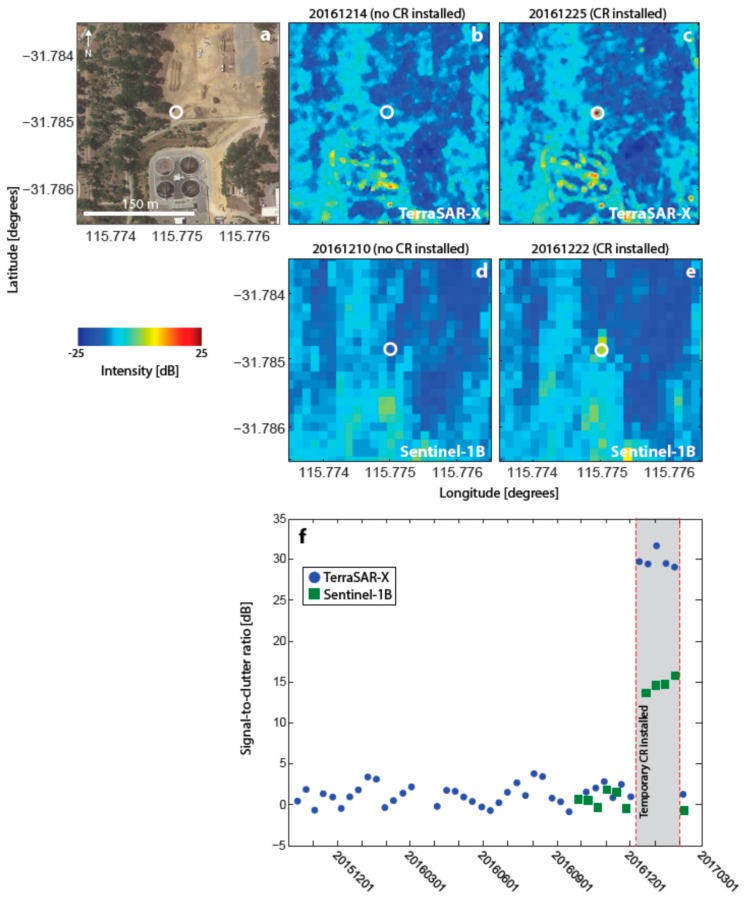
Corner reflector (CR) test installation at Beenyup wastewater injection plant (BEEN in Figure 1) as observed by both TSX and Sentinel-1B. (**a**) Google Earth view of the site with the CR location shown by the white circle. (**b**) TSX georeferenced backscatter intensity image prior to CR installation. (**c**) Same for (b) but after CR installation. (**d**) Sentinel-1B georeferenced backscatter intensity image prior to CR installation. (**e**) Same as for (d) but after CR installation. (**f**) Time series of signal-to-clutter ratio (SCR) at the location of the CR. Red lines and grey boxes indicate times when the CR was deployed.

Prior to the test deployment period at Beenyup, the Sentinel-1B satellite began to acquire and release SAR imagery over Perth. Therefore, backscatter intensity imagery from both TSX and Sentinel-1B could be analysed simultaneously to select a location for the CR test installation. The CR was deployed for four overpasses of both satellites (Table 2). Like PERT, this site is characterised by few other man-made structures and low levels of background clutter (−12 dB for both TerraSAR-X and Sentinel-1B). Figure 7 shows that the CR is visible in intensity imagery for both missions with SCRs of ~31 dB for TSX and ~15 dB for Sentinel-1B.

## 5. Discussion

### 5.1. Recommendations

**Figure 8:**
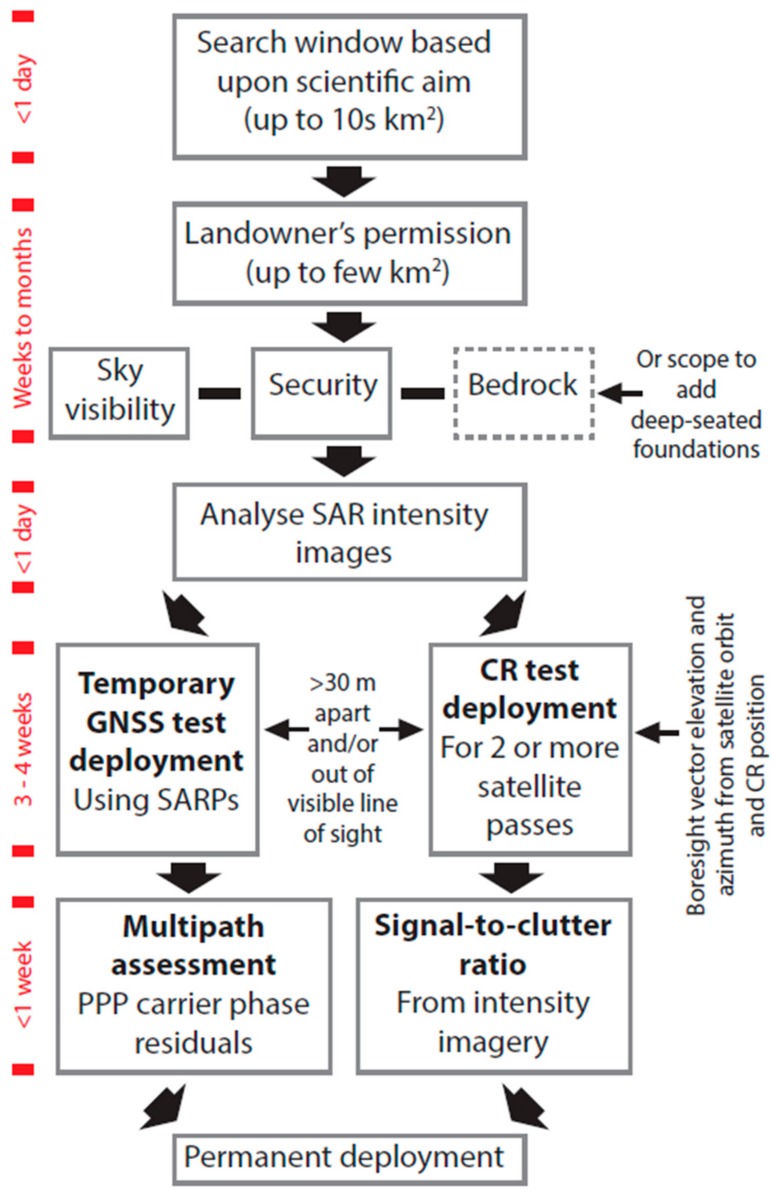
Flowchart of the recommended procedures for determining suitable sites for co-located cGNSS and corner reflectors (CRs) for integrated operational monitoring of vertical land motion (VLM). The time line provides a guide as to the time that should be allowed for each step, assuming that the user has access to, and experience with, the SAR and cGNSS data and processing tools. SARPs—standards and recommended practices.

The test experiments and procedure described here may be of use to others wishing to install co-located cGNSS and InSAR CR ground infrastructure for operational integrated monitoring of VLM elsewhere. Figure 8 shows a flowchart of our recommended procedures, and the approximate time taken to complete each step of the installation. 

In short, test co-locations of GNSS and CRs, followed by analysis of GNSS LC carrier-phase residuals (from PPP processing) and SCRs (from backscatter intensity imagery) are used to efficiently identify suitable sites before the installation of permanent ground infrastructure. Our primary concern was GNSS multipath caused by the co-located CR. We managed this by placing the CR at least ~30 m from the GNSS and not in visible line of sight. This strategy was vindicated by the test installations, and later confirmed after the permanent installations. On average, installation of a CR at the sites in this study resulted in SCRs of ~29 dB for TSX and ~13 dB for Sentinel-1B. These values are commensurate with SCRs observed for CRs installed in natural environments with X- and C-band SAR satellites [30,44], and suggests that our CRs are well placed for calibration of both datasets.

### 5.2. Integrating InSAR and cGNSS to Determine VLM

The advantages and disadvantages of the two techniques discussed in this paper are described elsewhere in the literature [24,65], including the factors that affect measurement accuracy, and the relative biases of each approach. Preliminary comparisons of the vertical displacement at HIL1 (for location, see Figure 1) relative to PERT from cGNSS and InSAR [41] indicate that there is consistency between the two measurement types in Perth, with displacements from Sentinel-1 and TSX within error of those recorded by cGNSS. As described in Section 1, we will be able to integrate measurements from both SAR missions with cGNSS, using the cGNSS-derived VLM to convert the relative InSAR-derived VLM to absolute values via the co-located CRs. This will be the focus of future studies, as some period of time must first elapse to allow collection of sufficient data to determine a reliable estimate of the VLM. In saying “some period of time”, we are being deliberatively speculative about how long this should be, as this appears to remain an open question, which depends on both the measurement precision and the magnitude of the VLM. 

One consideration for cGNSS-derived VLM demonstrated by [66] is that a period of at least 2.5 years is the minimum time span needed in the presence of annual signals, depending on the relative amplitudes of the linear and seasonal signals. He et al. [67] review other factors that can affect the precision of cGNSS-derived VLM. These, and primary citations, comprise reference frame stability [14], undetected offsets [68], seasonal loading models [69], common-mode errors [70], choice of noise model [57], choice of time series analysis software [61], and choice of GPS processing software and strategies [71]. An additional factor not included in [67] that affects GPS-derived VLM estimation is multipath [50] (Section 3.2).

The accuracy of the VLM estimate from InSAR is primarily a function of the frequency of acquisitions, the length of the observation period, and the magnitude of changes in atmospheric refraction [72,73], the latter of which is the largest source of measurement error. (Note that, because of the shorter instrument wavelength, λ ≈ 3.1 cm compared to λ ≈ 5.6 cm, X-band InSAR data experience greater dispersion due to the atmosphere than C-band, therefore TSX data are expected to be more sensitive to atmospheric variations than Sentinel-1B.) For the orbital repeat intervals of TSX and Sentinel-1B (11 and 12 days, respectively), [41] estimate that it would take around one year to detect the most recently published rate of VLM at cGNSS HIL1 (−3.12 ± 0.92 mm/year between 2005 and 2012: [40]). However, this estimate is likely to be optimistic as it does not account for seasonal effects. As such, we leave this to be determined by future research after we have acquired a longer time series of data.

In order to combine the cGNSS and InSAR measurements, the simplest approach will be to use a 1D polynomial [74,75], referencing all pixels to a CR proximal to a cGNSS from which the absolute VLM can be estimated and used to transform the InSAR data. Alternatively, higher order polynomials may be used to reduce the differences between the VLM estimated at the cGNSS and CRs across the network [3]. Interpolation of the cGNSS observations onto the same spatial grid as InSAR (using, e.g., Kriging or its geodetic counterpart, least squares prediction [76]), followed by optimisation may be used to determine the (full 3D) displacement field. This approach has been successful where cGNSS geodetic networks contain an order of magnitude more cGNSS stations than are available in Perth [9,77], and/or broad-scale levelling measurements [78]. Once the VLM estimates have been integrated into a consistent reference frame, the results from our network in Perth may be placed for integration into global analysis of coastal subsidence and sea level change.

### 5.3. Scope for Future Deployments

The co-located installations of ground infrastructure in Perth described here have been dictated by the existing locations of cGNSS on deep-seated pillars (PERT and STLG), the desire to determine VLM at tide gauges to correct the tide gauge record [40] (FMTL), and proximity to managed wastewater injection back into exploited aquifers (BEEN). The number of permanent sites has also been constrained by cost. If resources become available to install more sites, it will be possible to use the expected pattern of ground deformation inferred from groundwater drawdown measured at artesian monitoring bores, geophysical modelling [79], or reconnaissance InSAR studies [41] to determine the optimum spatial sampling of CRs and cGNSS in different settings [80]. A prime candidate site for another CR installation is the existing co-located cGNSS and tide gauge at Hillarys (HIL1 in Figure 1 and Table 1).

Finally, when active SAR transponders [46,81] become commercially available and can be operated legally within Australian signal transmission restrictions, we intend to explore further co-locations of transponders at these (and other) sites. This extends to the additional consideration of co-located DORIS beacons [23,82].

## 6. Conclusions

Using the example of Perth, Australia, we have described the practical considerations necessary for installing ground-based infrastructure to monitor VLM using cGNSS and SAR satellites. We present a series of recommendations that readers may adopt during the installation process, including the use of SCRs calculated from SAR intensity imagery to install CRs in regions of low background clutter, and tests on the spatial separation of the CR from the cGNSS to reduce multipath effects. In Perth, these installations will facilitate the integration of InSAR and cGNSS measurements to measure sub-cm scale VLM in an absolute reference frame. This will aid in our efforts to (1) constrain VLM in response to groundwater extraction and managed wastewater recharge, and (2) measure the impact of VLM upon the sea level record from local tide gauges.

## Figures and Tables

**Table 1:** Existing and new cGNSS infrastructure in the Perth region that was operating during the period of, and is also described in, this study (cf. Figure 1). GA—Geoscience Australia; RTKNetWest: http://www.rtknetwest.com.au/.

Name	Type	Local Operator	Monument	Receiver Type	Antenna Type	Longitude	Latitude
PERT	Existing (scientific)	GA	Deep-seated pillar	Trimble NetR9	TRM59800.00 NONE	115.885	−31.802
STLG	Existing (commercial)	RTKNetWest	Deep-seated pillar	Trimble NetR9	TRM55971.00 NONE	115.819	−31.866
FMTL	New (scientific)	Curtin University	Deep-seated pillar	Trimble NetR9	TRM59800.00 NONE	115.755	−32.049
HIL1	Existing (scientific)	GA	On tide gauge	Leica GRX1200GGPRO	ASH701945C_M NONE	115.739	−31.826

**Table 2:** Co-located cGNSS and CR installations in the Perth region described in this study. Included are the dates of test/permanent CR deployment and the availability of SAR data.

Site	cGNSS Status	Test CR Deployment		Permanent CR Deployment	
		Dates	SAR Data Available	Date	SAR Data Available
PERT	**Existing** (installed 18 August 1993)	18 May–8 June 2016	TSX	3 November 2016	TSX Sentinel-1B
STLG	**Existing** (installed 1 October 2012)	Location 1: 23 November–10 December 2015	TSX	-	-
		Location 2: 18 May–8 June 2016	TSX	10 August 2016	TSX (Sentinel-1B ~45 days after installation)
FMTL	**Installed during this study** Test installation (7–11 December 2015) Permanent installation (9 July 2016)	18 March–27 April 2016	TSX	19 May 2016	TSX (Sentinel-1B ~135 days after installation)
BEEN	**Proposed/awaiting construction** Test installation (12–15 December 2016)	12 December 2016–27 January 2017	TSX Sentinel-1B	-	-

**Table 3:** Comparison of the GPS-only mean ellipsoidal heights (m) and the average of the daily RMS of the LC carrier-phase residuals (mm) measured directly before, during, and directly after test and permanent corner reflector (CR) installations. The time period used before and after the test installation is equal to the duration of the test (given in Table 2), and for the permanent installation a time period of 20 days is used.

Site		Test CR Installation			Permanent CR Installation	
		Before	During	After	Before	After
PERT	Height (m)	12.670	12.668	12.664	12.671	12.667
	LC residual (mm)	9.6	9.3	9.0	8.6	8.9
STLG location 1	Height (m)	−0.021	−0.019	−0.019	-	-
	LC residual (mm)	10.3	10.6	10.6	-	-
STLG location 2	Height (m)	−0.019	−0.019	−0.023	−0.021	−0.021
	LC residual (mm)	10.8	10.5	10.3	10.8	10.3

**Table 4:** Comparison of the GPS-only average of the daily RMS of the carrier-phase (LC) residuals (mm) measured over different time periods directly before and directly after permanent corner reflector (CR) installations. The dates of the permanent installations are given in Table 2.

Site	5 Days		10 Days		20 Days		40 Days	
	Before	After	Before	After	Before	After	Before	After
PERT	8.5	8.5	8.5	8.7	8.6	8.9	9.0	9.1
STLG location 2	11.9	9.9	11.0	10.2	10.8	10.6	10.7	10.1

## References

[1] Schöne T., Schön N., Thaller D. (2009). IGS tide gauge benchmark monitoring pilot project (TIGA): Scientific benefits. J. Geod..

[2] Bouin M.N., Wöppelmann G. (2010). Land motion estimates from GPS at tide-gauges: A geophysical evaluation. Geophys. J. Int..

[3] Bock Y., Melga D. (2016). Physical applications of GPS geodesy: A review. Rep. Prog. Phys..

[4] Massonnet D., Feigl K.L. (1998). Radar interferometry and its application to changes in the Earth’s surface. Rev. Geophys..

[5] Bürgmann R., Rosen P.A., Fielding E.J. (2000). Synthetic aperture radar interferometry to measure Earth’s surface topography and its deformation. Ann. Rev. Earth Planet. Sci..

[6] Hanssen R.F. (2001). Radar Interferometry: Data Interpretation and Error Analysis.

[7] Amelung F., Galloway D.L., Bell J.W., Zebker H.A., Laczniak R.J. (1999). Sensing the ups and downs of Las Vegas: InSAR reveals structural control of land subsidence and aquifer-system deformation. Geology.

[8] Wright T.J., Parsons B.E., Lu Z. (2004). Toward mapping surface deformation in three dimensions using InSAR. Geophys. Res. Lett..

[9] Gudmundsson S., Sigmundsson F., Carstensen J.M. (2002). Three-dimensional surface motion maps estimated from combined interferometric synthetic aperture radar and GPS data. J. Geophys. Res. Solid Earth.

[10] Bürgmann R., Hilley G., Ferretti A., Novali F. (2006). Resolving vertical tectonics in the San Francisco Bay Area from permanent scatterer InSAR and GPS analysis. Geology.

[11] Legrand J., Bergeot N., Bruyninx C., Wöppelmann G., Bouin M.N., Altamimi Z. (2010). Impact of regional reference frame definition on geodynamic interpretations. J. Geod..

[12] Bevis M., Brown A., Kendrick E. (2013). Devising stable geometrical reference frames for use in geodetic studies of vertical crustal motion. J. Geod..

[13] Bevis M., Brown A. (2014). Trajectory models and reference frames for crustal motion geodesy. J. Geod..

[14] Griffiths J., Ray J. (2016). Impacts of GNSS position offsets on global frame stability. Geophys. J. Int..

[15] Finnegan N.J., Pritchard M.E., Lohman R.B., Lundgren P.R. (2008). Constraints on surface deformation in the Seattle, WA, urban corridor from satellite radar interferometry time-series analysis. Geophys. J. Int..

[16] Schmidt D.A., Bürgmann R. (2003). Time-dependent land uplift and subsidence in the Santa Clara valley, California, from a large interferometric synthetic aperture radar data set. J. Geophys. Res. Solid Earth.

[17] Parker A.L., Biggs J., Lu Z. (2016). Time-scale and mechanism of subsidence at Lassen Volcanic Center, CA, from InSAR. J. Volc. Geotherm. Res..

[18] Bock Y., Wdowinski S., Ferretti A., Novali F., Fumagalli A. (2012). Recent subsidence of the Venice Lagoon from continuous GPS and interferometric synthetic aperture radar. Geochem. Geophys. Geosyst..

[19] Dheenathayalan P., Small D., Schubert A., Hanssen R.F. (2016). High-precision positioning of radar scatterers. J. Geod..

[20] Gisinger C., Willberg M., Balss U., Klügel T., Mähler S., Pail R., Eineder M. (2017). Differential geodetic stereo SAR with TerraSAR-X by exploiting small multi-directional radar reflectors. J. Geod..

[21] Ferretti A., Savio G., Barzaghi R., Borghi A., Musazzi S., Novali F., Prati C., Rocca F. (2007). Submillimeter accuracy of InSAR time series: Experimental validation. IEEE Trans. Geosci. Remote Sens..

[22] Wöppelmann G., Le Cozannet G., de Michele M., Raucoules D., Cazenave A., Garcin M., Hanson S., Marcos M., Santamaría-Gómez A. (2013). Is land subsidence increasing the exposure to sea level rise in Alexandria, Egypt?. Geophys. Res. Lett..

[23] Raucoules D., Le Cozannet G., Wöppelmann G., de Michele M., Gravelle M., Daag A., Marcos M. (2013). High nonlinear urban ground motion in Manila (Philippines) from 1993 to 2010 observed by DInSAR: Implications for sea-level measurement. Remote Sens. Environ..

[24] Teatini P., Tosi L., Strozzi T., Carbognin L., Wegmuller U., Rizzetto F. (2005). Mapping regional land displacements in the Venice coastland by an integrated monitoring system. Remote Sens. Environ..

[25] Hung W.-C., Hwang C., Chen Y.-A., Chang C.-P., Yen J.-Y., Hooper A., Yang C.-Y. (2011). Surface deformation from persistent scatterers SAR interferometry and fusion with leveling data: A case study over the Choushui River alluvial fan, Taiwan. Remote Sens. Environ..

[26] Gray A.L., Vachon P.W., Livingstone C.E., Lukowski T.I. (1990). Synthetic aperture radar calibration using reference reflectors. IEEE Trans. Geosci. Remote Sens..

[27] Xia Y., Kaufmann H., Guo X.F. (2004). Landslide monitoring in the Three Gorges area using D-InSAR and corner reflectors. Photogramm. Eng. Remote Sens..

[28] Xia Y. CR-based SAR-interferometry for landslide monitoring. Proceedings of the IEEE International Geoscience and Remote Sensing Symposium.

[29] Froese C., Poncos V., Skirrow R., Mansour M., Martin D. Characterizing complex deep seated landslide deformation using corner reflector InSAR (CR-INSAR): Little Smoky Landslide, Alberta. Proceedings of the 4th Canadian Conference on Geohazards.

[30] Strozzi T., Teatini P., Tosi L., Wegmüller U., Werner C. (2013). Land subsidence of natural transitional environments by satellite radar interferometry on artificial reflectors. J. Geophys. Res. Earth Surf..

[31] Qin Y., Perissin D., Lei L. (2013). The design and experiments on corner reflectors for urban ground deformation monitoring in Hong Kong. Int. J. Antenn. Propag..

[32] Crosetto M., Gili J.A., Monserrat O., Cuevas-Gonzalez M., Corominas J., Serral D. (2013). Interferometric SAR monitoring of the Vallcebre landslide (Spain) using corner reflectors. Nat. Hazards. Earth Syst..

[33] Singleton A., Li Z., Hoey T., Muller J.-P. (2014). Evaluating sub-pixel offset techniques as an alternative to DInSAR for monitoring episodic landslide movements in vegetated terrain. Remote Sens. Environ..

[34] Fu W., Guo H., Tian Q., Guo X. (2010). Landslide monitoring by corner reflectors differential interferometry SAR. Int. J. Remote Sens..

[35] Garthwaite M.C., Hazelwood M., Nancarrow S., Hislop A., Dawson J.H. (2015). A regional geodetic network to monitor ground surface response to resource extraction in the northern Surat Basin, Queensland. Aust. J. Earth Sci..

[36] Marinkovic P., Larsen Y., Levinsen J.F., Broge N.H., Sørensen C.S., Dehls J. Something is “subsiding” in the state of Denmark-Operational prospects for nationwide subsidence mapping with Sentinel-1. Proceedings of the ESA Living Planet Symposium.

[37] Levinsen J.F., Broge N.H., Sørensen C.S. Approaching target: A service for nationwide deformation monitoring in Denmark using Sentinel-1. Proceedings of the Fringe 2017 10th International Workshop on Advances in the Science and Applications of SAR Interferometry and Sentinel-1 InSAR.

[38] Oyen A. Towards an InSAR based nationwide monitoring strategy in the Netherlands. Proceedings of the Fringe 2017 10th International Workshop on Advances in the Science and Applications of SAR Interferometry and Sentinel-1 InSAR.

[39] Featherstone W.E., Filmer M.S., Penna N.T., Morgan L.M., Schenk A. (2012). Anthropogenic land subsidence in the Perth Basin: Challenges for its retrospective geodetic detection. J. R. Soc. West. Aust..

[40] Featherstone W.E., Penna N.T., Filmer M.S., Williams S.D.P. (2015). Nonlinear subsidence at Fremantle, a long-recording tide gauge in the Southern Hemisphere. J. Geophys. Res. Oceans.

[41] Parker A.L., Filmer M.S., Featherstone W.E. (2017). First results from Sentinel-1A InSAR over Australia: Application to the Perth Basin. Remote Sens..

[42] Werninghaus R., Buckreuss S. (2010). The TerraSAR-X mission and system design. IEEE Trans. Geosci. Remote Sens..

[43] Torres R., Snoeij P., Geudtner D., Bibby D., Davidson M., Attema E., Potin P., Rommen B., Floury N., Brown M. (2012). GMES Sentinel-1 mission. Remote Sens. Environ..

[44] Garthwaite M.C. (2017). On the design of radar corner reflectors for deformation monitoring in multi-frequency InSAR. Remote Sens..

[45] Dow J.M., Neilan R.E., Rizos C. (2009). The international GNSS Service in a changing landscape of Global Navigation Satellite Systems. J. Geod..

[46] Mahapatra P., van der Marel H., van Leijen F., Samiei-Esfahany S., Klees R., Hanssen R. (2017). InSAR datum connection using GNSS-augmented radar transponders. J. Geod..

[47] Current IGS Site Guidelines. http://kb.igs.org/hc/en-us/articles/202011433-Current-IGS-Site-Guidelines.

[48] Guideline for Continuously Operating Reference Stations Special Publication 1. http://www.icsm.gov.au/publications/sp1/Guideline-for-Continuously-Operating-Reference-Stations_v2.1.pdf.

[49] Park K.-D., Nerem R.S., Schenewerk M.S., Davis J.L. (2004). Site-specific multipath characteristics of global IGS and CORS GPS sites. J. Geod..

[50] King M.A., Watson C.S. (2010). Long GPS coordinate time series: Multipath and geometry effects. J. Geophys. Res. Solid Earth.

[51] Fuhrmann T., Luo X., Knöpfler A., Mayer M. (2015). Generating statistically robust multipath stacking maps using congruent cells. GPS Solut..

[52] Atkins C., Ziebart M. (2016). Effectiveness of observation-domain sidereal filtering for GPS precise point positioning. GPS Solut..

[53] Standards and Practices for Control Surveys V1.7. http://www.icsm.gov.au/publications/sp1/sp1v1-7.pdf.

[54] Zumberge J.F., Heflin M.B., Jefferson D.C., Watkins M.M., Webb F.H. (1997). Precise point positioning for the efficient and robust analysis of GPS data from large networks. J. Geophys. Res. Solid Earth.

[55] Petit G., Luzum B. (2010). IERS Conventions.

[56] Boehm J., Werl B., Schuh H. (2006). Troposphere mapping functions for GPS and very long baseline interferometry from European Centre for Medium-Range Weather Forecasts operational analysis data. J. Geophys. Res. Solid Earth.

[57] Amos C.B., Audet P., Hammond W.C., Burgmann R., Johanson I.A., Blewitt G. (2014). Uplift and seismicity driven by groundwater depletion in central California. Nature.

[58] Hammond W.H., Blewitt G., Kreemer C. (2016). GPS imaging of vertical land motion in California and Nevada: Implications for Sierra Nevada uplift. J. Geophys. Res. Solid Earth.

[59] Devoti R., D’Agostino N., Serpelloni E., Pietrantonio G., Riguzzi F., Avallone A., Cavaliere A., Cheloni D., Cecere G., D’Ambrosio C. (2017). A combined velocity field of the Mediterranean region. Ann. Geophys..

[60] Systems Tool Kit. http://www.agi.com/products/stk/.

[61] Garthwaite M.C., Nancarrow S., Hislop A., Thankappan A., Dawson J.H., Lawrie S. (2015). The Design of Radar Corner Reflectors for the Australian Geophysical Observing System.

[62] Freeman A. (1992). SAR calibration: An overview. IEEE Trans. Geosci. Remote Sens..

[63] Bevis M., Scherer W., Merrifield M. (2002). Technical issues and recommendations related to the installation of continuous GPS stations at tide gauges. Mar. Geod..

[64] Shirzaei M., Ellsworth W.L., Tiampo K.F., González P.J., Manga M. (2016). Surface uplift and time-dependent seismic hazard due to fluid injection in eastern Texas. Science.

[65] Muller C., del Potro R., Biggs J., Gottsmann J., Ebmeier S.K., Guillaume S., Cattin P.-H., van der Laat R. (2014). Integrated velocity field from ground and satellite geodetic techniques: Application to Arenal volcano. Geophys. J. Int..

[66] Blewitt G., Lavallée D. (2002). Effect of annual signals on geodetic velocity. J. Geophys. Res. Solid Earth.

[67] He X., Montillet J.-P., Fernandes R., Bos M., Yu K., Hua X., Jiang W. (2017). Review of current GPS methodologies for producing accurate time series and their error sources. J. Geodyn..

[68] Gazeaux J., Williams S., King M., Bos M., Dach R., Deo M., Moore A.W., Ostini L., Petrie E., Roggero M. (2013). Detecting offsets in GPS time series: First results from the detection of offsets in GPS experiment. J. Geophys. Res. Solid Earth.

[69] Santamaría-Gomez A., Mémin A. (2015). Geodetic secular velocity errors due to interannual surface loading deformation. Geophys. J. Int..

[70] Serpelloni E., Faccenna C., Spada G., Dong D., Williams S.P.D. (2013). Vertical GPS ground motion rates in the Euro-Mediterranean region: New evidence of velocity gradients at different spatial scales along the Nubia-Eurasia plate boundary. J. Geophys. Res. Solid Earth.

[71] Bos M., Bastos L., Fernandes R.M.S. (2010). The influence of seasonal signals on the estimation of the tectonic motion in short continuous GPS time-series. J. Geodyn..

[72] Emardson T.R., Simons M., Webb F.H. (2003). Neutral atmospheric delay in interferometric synthetic aperture radar applications: Statistical description and mitigation. J. Geophys. Res. Solid Earth.

[73] Parker A.L., Biggs J., Walters R.J., Ebmeier S.K., Wright T.J., Teanby N.J., Lu Z. (2015). Systematic assessment of atmospheric uncertainties for InSAR data at volcanic arcs using large-scale atmospheric models: Application to the Cascade volcanoes, United States. Remote Sens. Environ..

[74] Zerbini S., Richter B., Rocca F., van Dam T., Matonti F. (2007). A Combination of space and terrestrial geodetic techniques to monitor land subsidence: Case Study, the southeastern Po Plain, Italy. J. Geophys. Res. Solid Earth.

[75] Papoutsis I., Papanikolaou X., Floyd M., Ji K.H., Kontoes C., Paradissis D., Zacharis V. (2013). Mapping inflation at Santorini volcano, Greece, using GPS and InSAR. Geophys. Res. Lett..

[76] Moritz H. (1980). Advanced Physical Geodesy.

[77] Wei M., Sandwell D., Smith-Konter B. (2010). Optimal combination of InSAR and GPS for measuring interseismic crustal deformation. Adv. Space Res..

[78] Fuhrmann T., Caro Cuenca M., Knöpfler A., van Leijan F.J., Mayer M., Westerhaus M., Hanssen R.F., Heck B. (2015). Estimation of small surface displacements in the Upper Rhine Graben area from a combined analysis of PS-InSAR, levelling and GNSS data. Geophys. J. Int..

[79] Brown N.J., Woods A.W., Neufeld J.A., Richardson C. (2014). Constraining Surface Deformation Predictions Resulting from Coal Seam Gas Extraction.

[80] Mahapatra P.S., Samiei-Esfahany S., Hanssen R.F. (2015). Geodetic network design for InSAR. IEEE Trans. Geosci. Remote Sens..

[81] Mahapatra P., Samiei-Esfahany S., van der Marel H., Hanssen R. (2014). On the use of transponders as coherent radar targets for SAR interferometry. IEEE Trans. Geosci. Remote Sens..

[82] Willis P., Fagard H., Ferrage P., Lemoine F.G., Noll C.E., Noomen R., Otten M., Ries J.C., Rothacher M., Soudarin L. (2010). The International DORIS Service: Toward maturity. Adv. Space Res..

